# Carbon Monoxide (CO) Released from Tricarbonyldichlororuthenium (II) Dimer (CORM-2) in Gastroprotection against Experimental Ethanol-Induced Gastric Damage

**DOI:** 10.1371/journal.pone.0140493

**Published:** 2015-10-13

**Authors:** Katarzyna Magierowska, Marcin Magierowski, Magdalena Hubalewska-Mazgaj, Juliusz Adamski, Marcin Surmiak, Zbigniew Sliwowski, Slawomir Kwiecien, Tomasz Brzozowski

**Affiliations:** 1 Department of Physiology, Jagiellonian University Medical College, Cracow, Poland; 2 Department of Forensic Toxicology, Institute of Forensic Research, Cracow, Poland; Temple University, UNITED STATES

## Abstract

The physiological gaseous molecule, carbon monoxide (CO) becomes a subject of extensive investigation due to its vasoactive activity throughout the body but its role in gastroprotection has been little investigated. We determined the mechanism of CO released from its donor tricarbonyldichlororuthenium (II) dimer (CORM-2) in protection of gastric mucosa against 75% ethanol-induced injury. Rats were pretreated with CORM-2 30 min prior to 75% ethanol with or without 1) non-selective (indomethacin) or selective cyclooxygenase (COX)-1 (SC-560) and COX-2 (celecoxib) inhibitors, 2) nitric oxide (NO) synthase inhibitor L-NNA, 3) ODQ, a soluble guanylyl cyclase (sGC) inhibitor, hemin, a heme oxygenase (HO)-1 inductor or zinc protoporphyrin IX (ZnPPIX), an inhibitor of HO-1 activity. The CO content in gastric mucosa and carboxyhemoglobin (COHb) level in blood was analyzed by gas chromatography. The gastric mucosal mRNA expression for HO-1, COX-1, COX-2, iNOS, IL-4, IL-1β was analyzed by real-time PCR while HO-1, HO-2 and Nrf2 protein expression was determined by Western Blot. Pretreatment with CORM-2 (0.5–10 mg/kg) dose-dependently attenuated ethanol-induced lesions and raised gastric blood flow (GBF) but large dose of 100 mg/kg was ineffective. CORM-2 (5 mg/kg and 50 mg/kg i.g.) significantly increased gastric mucosal CO content and whole blood COHb level. CORM-2-induced protection was reversed by indomethacin, SC-560 and significantly attenuated by celecoxib, ODQ and L-NNA. Hemin significantly reduced ethanol damage and raised GBF while ZnPPIX which exacerbated ethanol-induced injury inhibited CORM-2- and hemin-induced gastroprotection and the accompanying rise in GBF. CORM-2 significantly increased gastric mucosal HO-1 mRNA expression and decreased mRNA expression for iNOS, IL-1β, COX-1 and COX-2 but failed to affect HO-1 and Nrf2 protein expression decreased by ethanol. We conclude that CORM-2 released CO exerts gastroprotection against ethanol-induced gastric lesions involving an increase in gastric microcirculation mediated by sGC/cGMP, prostaglandins derived from COX-1, NO-NOS system and its anti-inflammatory properties.

## Introduction

Carbon monoxide (CO) is a gaseous molecule generated intracellularly during the degradation of heme, a product of iron protoporphyrin IX, in reaction catalyzed by heme oxygenase (HO) in the presence of molecular oxygen and reducing equivalents (NADPH) [1]. So far three isozymes of HO have been identified [2]. The first isoform of HO called HO-1 is inducible by a large number of stressful stimuli, such as heavy metals, UV radiation, hypoxia, hyperoxia, infections, heme own substrates and hemoglobins [3]. Isoform HO-2 is constitutively expressed in most tissues while a third HO, recently discovered isoform HO-3, has, as yet, unknown function [4]. Additionally, it has been proven that production of small amounts of CO from heme independent sources could originate from lipid peroxidation, xenobiotics and the metabolic activity of intestinal bacteria [5].

Previous studies revealed that CO can modulate a variety of physiological processes, including vasodilatation, neurotransmission, platelet activation and aggregation [6]. It is nowadays accepted that CO exerts pleiotropic cellular effects by acting through a number of signaling pathways including those regulated by mitogen-activated protein kinases (MAPKs), peroxisome proliferator-activated receptor gamma (PPAR-γ), cellular ROS production, calcium-dependent potassium channels (Kca) and soluble guanylyl cyclase (sGC) [7, 8, 9]. Moreover, numerous studies have demonstrated that HO-1 and its metabolites, CO and biliverdin can modulate the inflammatory processes [10, 11]. CO, acting *via* MAPKs activity, inhibits the production of pro-inflammatory cytokines (TNF-α, IL-1β) *in vitro* and *in vivo* and increases the expression of the anti-inflammatory cytokine IL-10. Not surprisingly, as a result of high affinity to hemoproteins such as cytochromes and NADPH oxidase, CO can downregulate production of reactive oxygen species (ROS) [12]. Based on these findings a novel class of compounds, termed CO-releasing molecules (CORMs), has been designed to liberate this gaseous molecule in variety of biological systems and to determine its effects in different organs [13]. CORMs were reported to serve as pharmacological tool to assess the involvement of CO in protection against various diseases due to anti-inflammatory action of CO-released from these compounds [13].

Growing body of evidence emphasize the important role of CO in the physiology and pathophysiology of gastrointestinal (GI) tract [14]. For instance, CO participates in the secretion of duodenal HCO_3_^-^ ions and the restoration of altered duodenal activity in diabetes [15, 16]. Furthermore, exogenously applied CO exerts potent anti-oxidative, anti-proliferative and anti-apoptotic properties [17]. Chronic HO-1 induction regulates metabolism in diabetes and obesity by restoration of insulin sensitivity and lowering body weight [18, 19]. CO has been reported to enhance bile secretion [20], play a pivotal role in hepatoprotection [21, 22] and attenuate the intestinal graft ischemia/reperfusion injury in rats [23].

Gaseous molecule CO appears to act as a significant component in complex mechanism of gastric mucosal defense, however, the contribution of this vasoactive mediator to the mechanism of gastroprotection against acute gastric lesions has been little elucidated. Therefore, in the present study, we examined the effect of an intragastric application of the tricarbonyldichlororuthenium (II) dimer (CO-releasing molecule; CORM-2), a CO donor, against necrotizing injury of gastric mucosa induced by 75% ethanol as well as the underlying mechanism of the potential protective activity of this gaseous mediator. In order to get insight into mechanism of action of CORM-2 in the stomach exposed to ethanol, rats with the inhibition of prostaglandins (PG) generation and the nitric oxide (NO) biosynthesis as well as those with inhibited cGMP and HO-1 activity have been used to determine whether PG/cyclooxygenase (COX), sGC/cGMP and NO/NO-synthase (NOS) systems are involved in gastroprotection exerted by CO released from CORM-2. We also attempted to determine the ability of CORM-2 to increase CO content in gastric mucosa and in whole blood as carboxyhemoglobin (COHb) level and to alter the mRNA or protein expression of HO-1, HO-2, Nrf2, COX-2, iNOS, proinflammatory and anti-inflammatory cytokines IL-1β and IL-4, respectively, in gastric mucosa compromised by 75% ethanol.

## Materials and Methods

### Animals, chemicals, drugs application and treatments

The total number of 230 male Wistar rats with weight averaging about 250 g was used in this study. Rats were fasted for 24 hours with free access to drinking water before the experiment. The study was approved by the Institutional Animal Care and Use Committee of Jagiellonian University Medical College in Cracow and run in accordance with the statements of the Helsinki Declaration regarding handling of experimental animals.

Rats were randomly selected into four major groups (A, B, C and D) each consisting of 6–8 animals and were pretreated i.g. 30 min before 75% ethanol (1 ml i.g.) application either with: 1) vehicle (saline; 1 ml/rat); 2) CORM-2 (Sigma-Aldrich, Schnelldorf, Germany) applied in graded doses ranging from 0.5 mg/kg up to 100 mg/kg and RuCl_3_ (5 mg/kg i.g., Sigma-Aldrich, Schnelldorf, Germany) as a negative control to CORM-2 [16] (series A). In series B, animals were pretreated 30 min before i.g. application of CORM-2 (5 mg/kg) or vehicle (saline) with the non-selective cyclooxygenase (COX) inhibitor indomethacin (5 mg/kg i.p., Sigma-Aldrich, Schnelldorf, Germany) or the selective COX-1 inhibitor, SC-560 (5 mg/kg i.g., Cayman Chemical, Ann Arbor, USA) or the selective COX-2 inhibitor, celecoxib (10 mg/kg i.g., Pfizer, Illertissen, Germany) in the absence or presence of synthetic analog of PGE_2_ (16,16 dimethyl PGE_2_, 5 μg/kg i.g., Sigma-Aldrich, Schnelldorf, Germany). Similarly as in series A, all rats of series B received 75% ethanol in a volume of 1 ml (i.g.) 30 min following CORM-2 or vehicle administration. Rats of series C were administered 30 min before i.g. vehicle (saline) or CORM-2 applied in standard dose of 5 mg/kg i.g. with the inhibitor of NOS, N^G^-nitro-L-arginine (L-NNA, 20 mg/kg i.g. Sigma-Aldrich, Schnelldorf, Germany), followed 30 min later by 75% ethanol as described for animals of series A and B. The separate group of these animals (series C) received L-arginine (200 mg/kg i.g. Sigma-Aldrich, Schnelldorf, Germany), a substrate for NOS in the presence of L-NNA with or without the combination with CORM-2 (5 mg/kg i.g.) and these rats also received 30 min later 75% ethanol (1 ml i.g.). Rats of series D were pretreated 30 min before CORM-2 (5 mg/kg i.g.) or vehicle (saline) administration with 1H-[1,2,4]oxadiazolo[4,3-a]quinoxalin-1-one (ODQ, 10 mg/kg i.p., Sigma-Aldrich, Schnelldorf, Germany), an inhibitor of sGC [24], or hemin (Sigma-Aldrich, Schnelldorf, Germany), the inductor of HO-1 activity [25] or zinc protoporphyrin IX (ZnPPIX, 5 mg/kg i.p. Sigma-Aldrich, Schnelldorf, Germany), an inhibitor of HO-1 activity [26], and following 30 min of CORM-2 or vehicle application, these rats received 75% ethanol (i.g.) as in case of animals of series A, B and C. Each pretreatment with COX-1, COX-2, NOS inhibitors, cGMP, HO-1 antagonists or hemin was administered 30 min prior to a subsequent application of CORM-2 followed 30 min later by i.g. application of 75% ethanol (i.g.) in the volume of 1.5 ml using orogastric tube to induce acute gastric mucosal lesions, as reported by our group previously [27].

### Determination of gastric blood flow and the area of gastric lesions

At the termination of each experiment one hour after i.g. application of 75% ethanol, animals were anesthetized with pentobarbital (60 mg/kg i.p.), their abdomens were opened and the stomachs were exposed to measure GBF by means of H_2_-gas clearance technique as described previously [28]. The GBF was measured in fundic part of the gastric mucosa not involving mucosal lesions. Average values of three measurements were determined and expressed as a percentage of change of the value determined in vehicle-control gastric mucosa. The area of gastric lesions in each rat stomach was determined with computerized planimetry (Morphomat, Carl Zeiss, Berlin, Germany) to blind control by the person who did not know to whom experimental group of animals belonged to [29, 30].

### Expression of mRNA for HO-1, COX-2, iNOS and IL-1β in the rat gastric mucosa determined by real time reverse transcriptase-polymerase chain reaction (qPCR)

Immediately after GBF measurement and assessment of area of gastric lesions, the gastric mucosal biopsies were quickly collected, snap-frozen, and stored at −80°C. RNA was isolated using GeneMATRIX Universal RNA Purification Kit, EURx, Gdansk, Poland and reversed trascription to cDNA was performed using High-Capacity cDNA Reverse Transcription Kit (Thermo Fisher Scientific, Life Technologies, MA, USA). Expression for HO-1, COX-2, iNOS and IL-1β was determined by real-time PCR (qPCR) using specific primers designed in Primer-BLAST (National Center for Biotechnology Information database), SYBR-Green including kit, SG qPCR Master Mix (2x) (EURx, Gdansk, Poland) and appropriate thermal cycler (7900HT Fast Real-Time PCR System, Thermo Fisher Scientific, Life Technologies, MA, USA). The nucleotide sequences of the primers used in PCR are presented in Table 1. β-actin gene was used as an internal control. Data was analysed using 2^-ΔCt^ method [31].

**Table 1:** Forward and reverse primers used in the assessment of mRNA expression for β-actin, HO-1 COX-2, iNOS and IL-103B2 by real time polymerase chain reaction (qPCR).

Gene	Forward primer	Reverse primer
***β-actin***	5’- GATCAAGATCATTGCTCCTCCTG -3’	5’- AGGGTGTAAAACGCAGCTCA -3’
***HO-1***	5’- GTCCCAGGATTTGTCCGAGG -3’	5’- GGAGGCCATCACCAGCTTAAA -3’
***COX-2***	5’- ATCAGAACCGCATTGCCTCT -3’	5’- GCCAGCAATCTGTCTGGTGA -3’
***iNOS***	5’- TGGTGAGGGGACTGGACTTT -3’	5’- CTCCGTGGGGCTTGTAGTTG -3’
***IL-1β***	5’- GCTATGGCAACTGTCCCTGA -3’	5’- AGTCAAGGGCTTGGAAGCAA -3’

### Protein expression of HO-1, HO-2 and Nrf2 in gastric mucosa pretreated with or without vehicle or CORM-2 determined by Western Blot

Western blot analysis was used to determine gastric expression of HO-1, HO-2, and Nrf2 in gastric mucosal samples obtained from intact rats and those with ethanol- induced gastric damages pretreated with vehicle or CORM-2 (1–100 mg/kg i.g.). Gastric mucosal specimens were homogenized and lysed in lysis buffer (50 mM Tris 7.5 pH, 130 mM NaCl, protease inhibitor cocktail (SIGMAFAST™ Protease Inhibitor Cocktail Tablets, Sigma Aldrich, Schnelldorf, Germany) and 1% NP-40. Proteins concentration was determined by Bradford assay (Sigma Aldrich, Schnelldorf, Germany). Proteins (60–80 μq per well) were separated on 8% polyacrylamide gels and electro-blotted onto PVDF membrane, subsequently blocked for 45 minutes in 5% non-fat milk. Mouse monoclonal anti-HO-1 (Santa Cruz, CA, USA) in dilution of 1:250 in 3% bovine serum albumin, rabbit polyclonal anti-HO-2 (Proteintech, Manchester, UK) in dilution of 1:500 in 5% milk, anti-Nrf2 (1:500, Santa Cruz, CA, USA) (incubation in 4°C, overnight) and anti-β-actin (Santa Cruz, CA, USA) in dilution of 1:2000 in 5% milk (incubation in room temperature, 3 h) were used as primary antibodies. Protein expression was visualized using a secondary goat anti-mouse IgG or goat anti-rabbit IgG antibodies in dilution of 1:2000 in 5% milk (incubation time 1 h, room temperature), where appropriate. Secondary antibodies were conjugated to horseradish peroxidase and enhanced chemiluminescence was measured using detection kit (WesternSure® ECL Substrate (LI-COR, NE, USA) on C-DiGit® Blot Scanner (LI-COR, NE, USA). The intensity of the bands was determined and analyzed using Image Studio 4.0 software (LI-COR, NE, USA). The expression of each sample was normalized to the expression of β-actin protein.

### Determination of CO content in gastric mucosa and blood samples by gas chromatography (GC)

In order to determine the CO concentration in gastric mucosa and blood samples, rats were treated i.g. with 1 ml of vehicle-control (saline) or CORM-2 applied i.g. in a standard protective dose of 5 mg/kg and higher dose of 50 mg/kg. After 30 minutes, animals were sacrificed, gastric mucosa samples and blood from *vena cava* were collected for the chromatographic assessment of CO content using modification of previously described method [32]. In brief, the principle of method used for CO determination was based on CO release from its connection with hemoglobin (Hb) molecule due to the change in the oxidation state of Fe ion located in the center of the porphyrin ring from +2 to +3 (which do not have the ability for binding CO). The analyte (CO) was then catalytically converted to CH_4_ and quantified by the use of flame ionization detector (FID). In our experiments, the gastric mucosa samples were transferred into 20 ml HS vials and tightly sealed. Thereafter, the volume of 1.0 ml of distilled water was added to each vial by means of syringe and the vials were placed into ultrasonic bath for about three hours for Hb extraction. To release CO, the water solution of K3[Fe(CN)6] in a volume of 1.0 ml of 10% (m/m) was added to the sealed vials. The vials were then shaken for 30 minutes (1000 rev/min) to achieve complete gas liberation, subjected later to GC analysis.

The detector response was converted into sample’s CO volume using linear calibration function obtained after analysis of standards containing different amounts of analyte. The standards were prepared by adding known volume of CO: 0.0, 0.5, 1.0, 2.0, 5.0, 10.0, 20.0 and 50.0 ml to the sealed HS vials which contained 2.0 ml of water. Standards for each calibration level were prepared in triplicate.

Determination of COHb in blood samples was performed as follows; the volume of 1 ml of whole blood was diluted with 9 ml of water and 50 mg of sodium dithionite was added to reduce methemoglobin (MetHb). After thorough mixing, three samples, each of 1 ml, were transferred into 20 ml headspace vials and tightly sealed. The remaining blood (7 ml) was saturated with CO in a test-tube and then flushed with N_2_. To minimize change of solution volume due to solvent evaporation, both CO and nitrogen, which was used to remove CO physically dissolved in solution (not bonded with Hb), were initially saturated with water. The saturated blood was then further 10-fold diluted and COHb standards containing 10.0%, 7.5%, 5.0%, 2.5% and 0.0% COHb were then prepared by appropriate dilution with water. To each standard, which was prepared in duplicate, the water solution of K_3_[Fe(CN)_6_] was also added in a volume of 1.0 ml of 10% (m/m) to release CO.

Chromatographic separation was achieved using column packed with molecular sieves in isothermal mode. Instrumental parameters of the used HS-GC-FID method were summarized in Table 2.

**Table 2:** HS-GC-FID method instrumental parameters.

Parameter	Value
thermostatic temperature	80°C
thermostatic time	10 min
needle temperature	100°C
capillary temperature	110°C
sample time analysis	5 min
injection time	0.04 min
injector temperature	240°C
carrier gas	N_2_, 32 ml/min
oven temperature	120°C
methanizer temperature	350°C
detector temperature	350°C

Autosystem XL gas chromatograph, controlled by Totalchrom Navigator 6.3.1 software, and equipped with HS 40 headspace analyser, a column packed with molecular sieve 5Å 80/100 Mesh (6’x1/8”) and FID, all manufactured by Perkin Elmer (Waltham, USA), was used in the study. The instrument was modified by incorporation of a methanizer unit packed with Ni catalyst between the columns end and the detector. This unit converts CO and CO_2_ into CH_4_, Vortex MS2 (IKA, Staufen, Germany) and Sonic 6D ultrasonic bath (Polsonic, Warsaw, Poland) were used in sample preparation step whereas 5.0 ml and 50.0 ml gastight syringes (Hamilton, Reno, USA) were used for standards preparation. Analytical grade K3[Fe(CN)6] was purchased from Avantor Performance Materials Poland (Gliwice, Poland). Working solutions were prepared using purified water obtained from NANOpure Diamond Barnstead system (Thermo Fisher Scientific, Waltham, USA). Nitrogen (99.99%) was generated in the laboratory using Nitrox Ltd. (GB) NDD FCN system. CO (chemically pure grade) was purchased from Airproducts (Allentown, USA).

### Statistical analysis

Results are expressed as mean ± SEM. Statistical comparison was performed by Student’s T-test or ANOVA with Tukey *post-hoc* test where appropriate. Difference with p <0.05 was considered significant. Data was analyzed using GraphPad Prism 5.0 Software.

## Results

**Fig 1:**
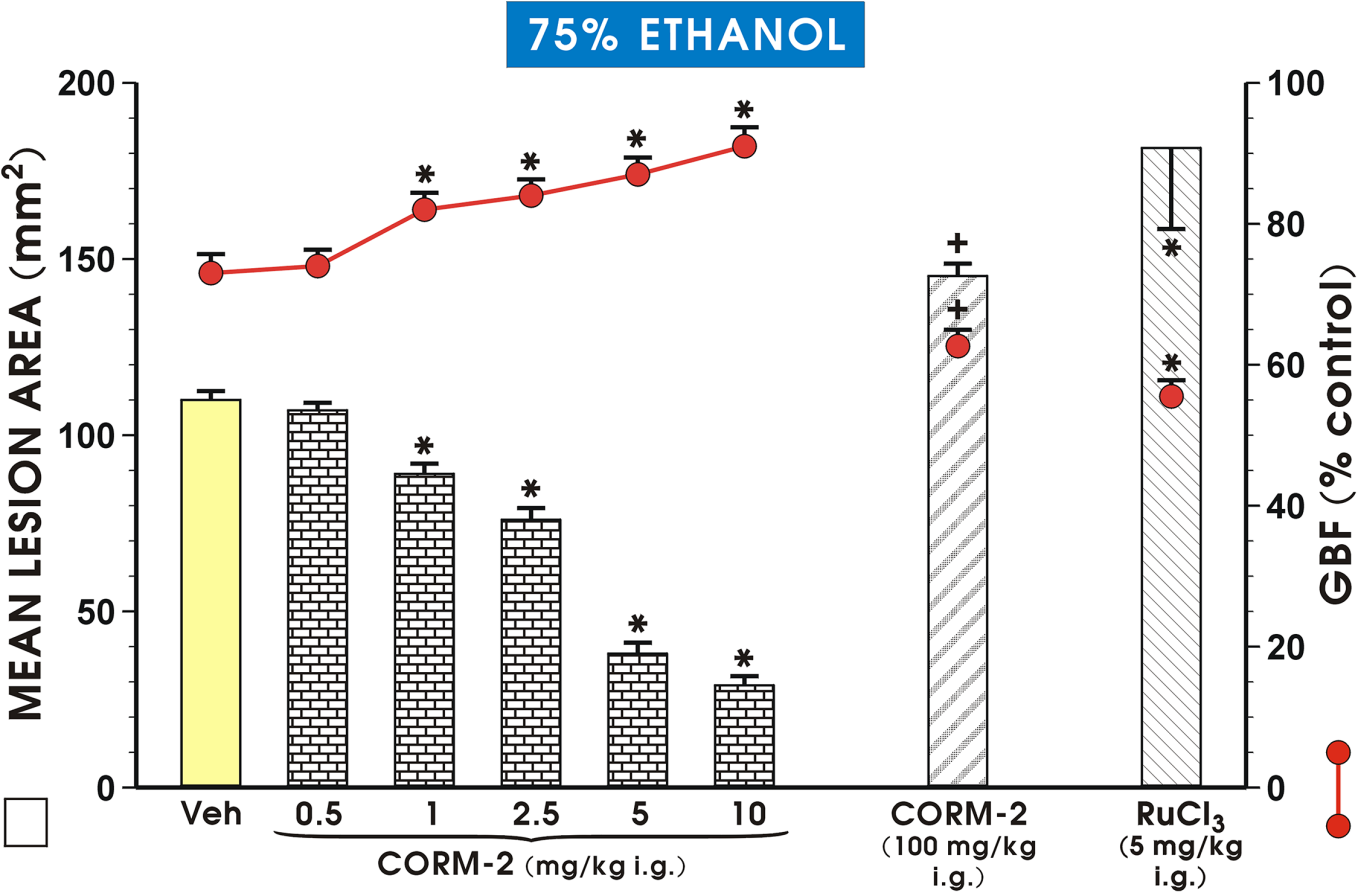
Mean lesion area of ethanol—induced gastric lesions and gastric blood flow (GBF) in the gastric mucosa of rats pretreated with CORM-2 applied in graded doses ranging from 0.5 mg/kg up to 10 mg/kg and treated 30 min later with 75% ethanol. For comparison the effect of pretreatment with CORM-2 applied i.g. in higher dose of 100 mg/kg is presented. Vehicle (Veh)-control group received 1 ml of saline (i.g.). Results are mean ± S.E.M. of 6–8 rats per group. Significant change (p<0.05) as compared with the respective values in Veh-control group is indicated by asterisk. Cross indicates significant change (p<0.05) as compared with Veh-control or CORM-2 (5 mg/kg i.g.) administered group.

Fig 1 shows that pretreatment with CORM-2 (0.5–10 mg/kg i.g.) dose-dependently decreased the mean area of ethanol-induced gastric lesion and significantly (p<0.05) increased the GBF as compared with vehicle-control group pretreated with saline (p<0.05). CORM-2 applied in a dose of 0.5 mg/kg i.g. failed to significantly affect the area of gastric lesions and the GBF, but the significant decrease in the mean lesion area and increase in the GBF (p<0.05) were observed starting from the dose of 1 mg/kg of CORM-2 and these effects were potentiated by CORM-2 administered in higher doses up to 10 mg/kg (Fig 1). The standard dose of CORM-2 inhibiting ethanol lesions by 50% (ID_50_) was 5 mg/kg and this dose of CORM-2 has been used in our subsequent determinations. As shown in Fig 1, CORM-2 administered i.g. in the higher dose of 100 mg/kg significantly increased the area of gastric mucosal injury caused by 75% ethanol (p<0.05) and significantly (p<0.05) decreased GBF as compared with those in vehicle-pretreated rats exposed to 75% ethanol. The administration of RuCl_3_ (5 mg/kg i.g.) as a negative control to CORM-2, which does not release CO [16] significantly (p<0.05) increased the area of gastric damage and significantly decreased (p<0.05) GBF level as compared with vehicle-control group.

As shown in Table 3, CORM-2 applied in a standard dose 5 mg/kg i.g. which significantly reduced ethanol-induced gastric lesions and raised the GBF (Fig 1) significantly increased (p<0.05) the concentration of CO in gastric mucosa as compared to respective value obtained in vehicle-treated rats. CORM-2 applied i.g. in the higher dose of 50 mg/kg significantly increased (p<0.05) CO content in gastric mucosa as compared to the respective values obtained in vehicle- and CORM-2 (5 mg/kg i.g.)- treated rats. Similarly, the blood level of COHb was significantly increased (p<0.001) in blood samples collected from rats treated with CORM-2 in a dose of 5 mg/kg i.g. as compared with vehicle-saline control. CORM-2 applied in the higher dose of 50 mg/kg i.g. significantly increased COHb level vs. vehicle-control (p<0.001) and CORM-2 (5 mg/kg i.g.) treated group (p<0.01).

**Table 3:** The CO content in gastric mucosa and COHb level in blood of rats treated i.g. with vehicle (saline) or CORM-2 (5 mg/kg or 50 mg/kg). Results are mean ± S.E.M of 3–5 determinations. Asterisk indicates significant change (p<0.05 or p<0.001) as compared with vehicle-control group. Double asterisk indicates significant change (p<0.05, p<0.01 or p<0.001) as compared with vehicle-control and CORM-2 (5 mg/kg i.g.) treated group.

Experimental group	CO in gastric mucosa [ml/g]	COHb in blood [% of total Hb]
***Vehicle***	1.040 ± 0.1077	0.7750 ± 0.025
***CORM-2 (5 mg/kg i*.*g*.*)***	1.900 ± 0.3464*	1.550 ± 0.1190*
***CORM-2 (50 mg/kg i*.*g*.*)***	3.675 ± 0.6142**	2.200 ± 0.1080**

**Fig 2:**
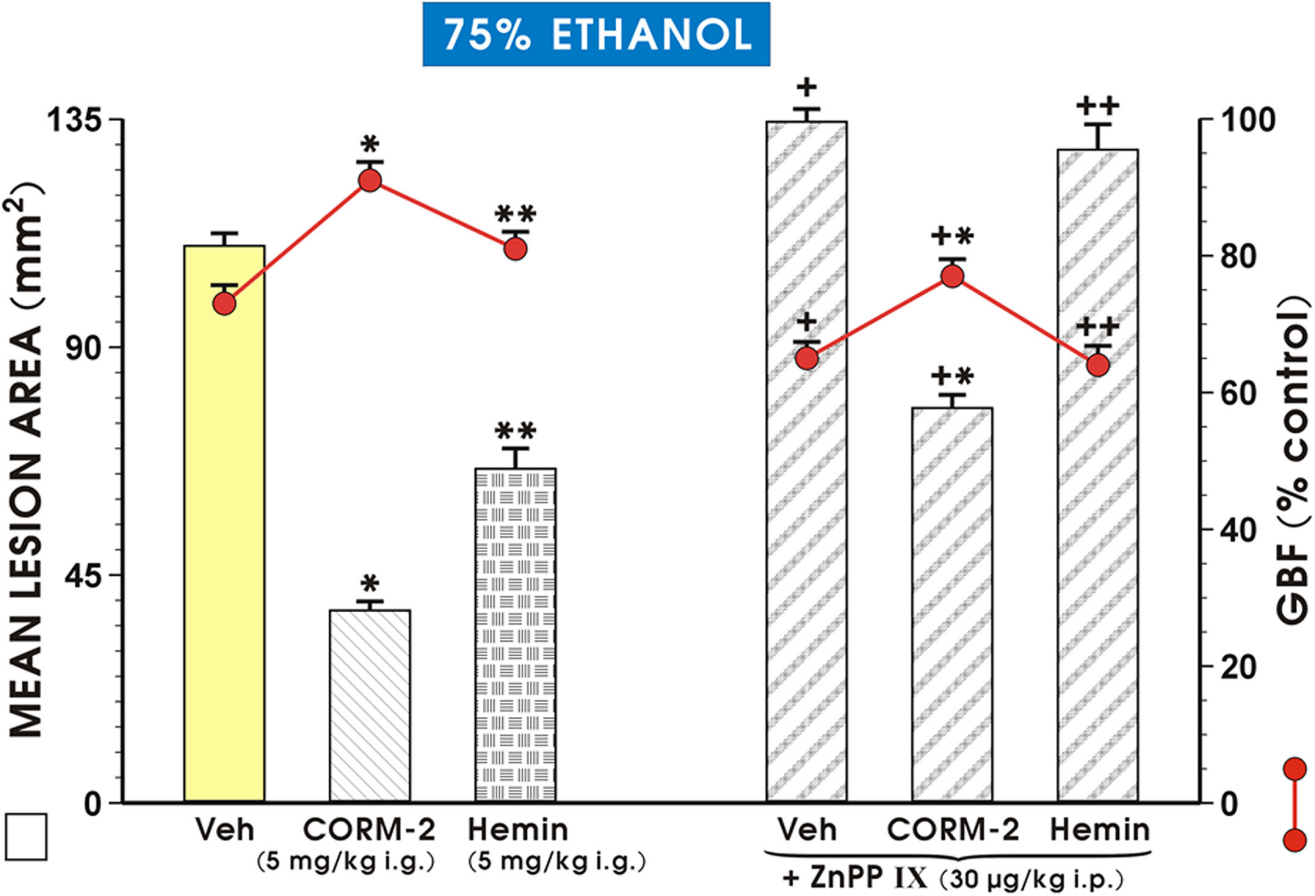
Mean lesion area of ethanol damage and the alteration in the gastric blood flow (GBF) in rats which were pretreated with zinc protoporphyrin IX (ZnPPIX) in the presence of Vehicle (Veh), CORM-2 (5 mg/kg i.g.) or hemin (5 mg/kg i.g.) and treated 30 min later with 75% ethanol. Vehicle (Veh)-control group received saline (1 ml i.g.). Results are mean ± S.E.M. of 6–8 rats per each group. The significant change (p<0.05) as compared with the respective values in Veh-control group was indicated by asterisk. Double asterisks indicate a significant change (p<0.05) as compared with the respective values obtained with CORM-2 (5 mg/kg i.g.) and vehicle-pretreated group. Cross indicates a significant change (p<0.05) comparing to the values obtained in rats pretreated with vehicle. Cross and asterisk indicate a significant change (p<0.05) as compared with the respective values in CORM-2-pretreated animals. Double crosses indicate a significant change (p<0.05) as compared with the respective values in hemin-pretreated animals.

Fig 2 shows that the pretreatment with CORM-2 (5 mg/kg i.g.) resulted in a similar reduction of the area of ethanol-induced gastric lesions and an increase in GBF as presented in Fig 1. The pretreatment with hemin (5 mg/kg i.g.) significantly (p<0.05) reduced ethanol-induced injury and significantly (p<0.05) increased GBF, though these alterations were less pronounced as in case of CORM-2 (Fig 2). The mean area of ethanol-induced gastric lesions was significantly increased in rats pretreated with ZnPPIX (30 mg/kg i.p.) as compared with vehicle-pretreated controls (p<0.05). The pretreatment with ZnPPIX significantly inhibited the reduction in the area of ethanol-induced gastric lesions and an increase in GBF caused by CORM-2 and hemin (p<0.05) (Fig 2).

**Fig 3:**
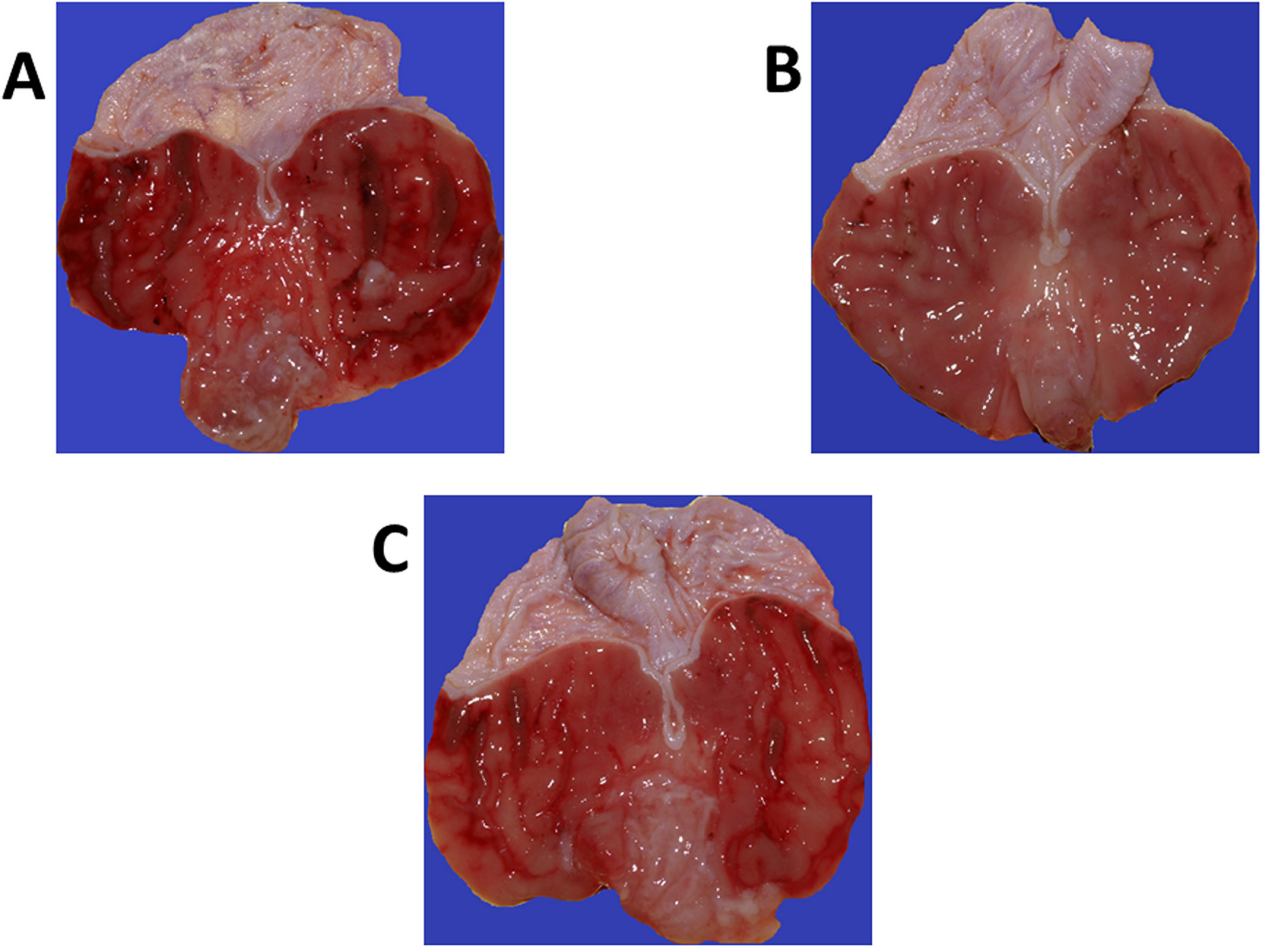
The representative gross appearance of 75% ethanol-induced gastric lesions in the rat stomach pretreated intragastrically (i.g.) with vehicle (saline) 30 min before the exposure to ethanol and sacrificed 1 h after ethanol application (A). Note, the presence of numerous band-like lesions mainly localized to oxyntic mucosa. In CORM-2 (5 mg/kg i.g) pretreated rat, the area of ethanol-induced gastric lesions was markedly reduced (B). The combination of zinc protoporphyrin (ZnPPIX, 30 μg/kg i.p.), the inhibitor of HO-1 activity, and CORM-2 (5 mg/kg i.g.) resulted in an increase in the area of ethanol-induced gastric lesions as compared with application of this CO donor alone (C vs. B).

The macroscopic appearance of the rat gastric mucosa pretreated with vehicle (saline) and CORM-2 (5 mg/kg i.g.) is presented in Fig 3A and 3B. In vehicle-pretreated rats, the numerous gastric hemorrhagic band-like lesions were observed but these lesions were markedly reduced by pretreatment with CORM-2 (5 mg/kg i.g.) (Fig 3A vs. Fig 3B). As shown in Fig 3C, ZnPPIX combined with CORM-2 reversed the decrease in the macroscopically assessed gastric lesions evoked by pretreatment with this CO donor (Fig 3C vs. Fig 3B).

**Fig 4:**
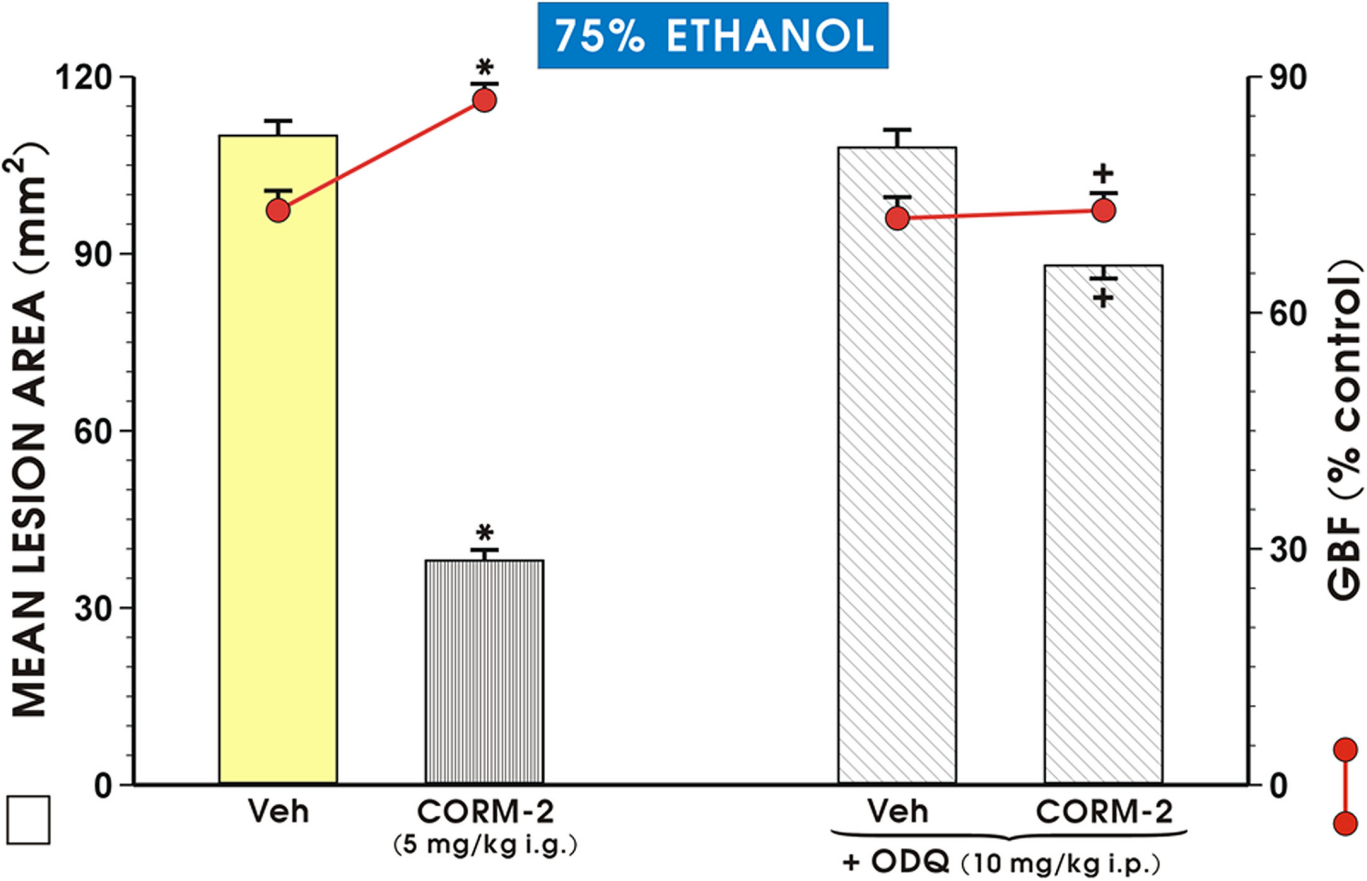
Mean lesion area of ethanol damage and the alteration in the gastric blood flow (GBF) in rats treated with ODQ in the presence of CORM-2 (5 mg/kg i.g.) and treated 30 min later with 75% ethanol. Vehicle (Veh)-control group was pretreated with saline (1 ml i.g.). Results are mean ± S.E.M. of 6–8 rats per each group. Significant change (p<0.05) as compared with the respective values in Veh-control group is indicated by the asterisk. Cross indicates a significant change (p<0.05) comparing to the values obtained in rats treated with CORM-2 alone.

As shown in Fig 4, the area of ethanol-induced gastric lesions and the GBF were not significantly different in rats pretreated with ODQ (10 mg/kg i.p.) alone as compared with vehicle (saline)-pretreated controls. However, the co-administration of ODQ with CORM-2 resulted in a significant increase in the mean lesion area and a significant decrease in the GBF (p<0.05) as compared with respective values obtained in rats pretreated with CORM-2 alone (Fig 4).

**Fig 5:**
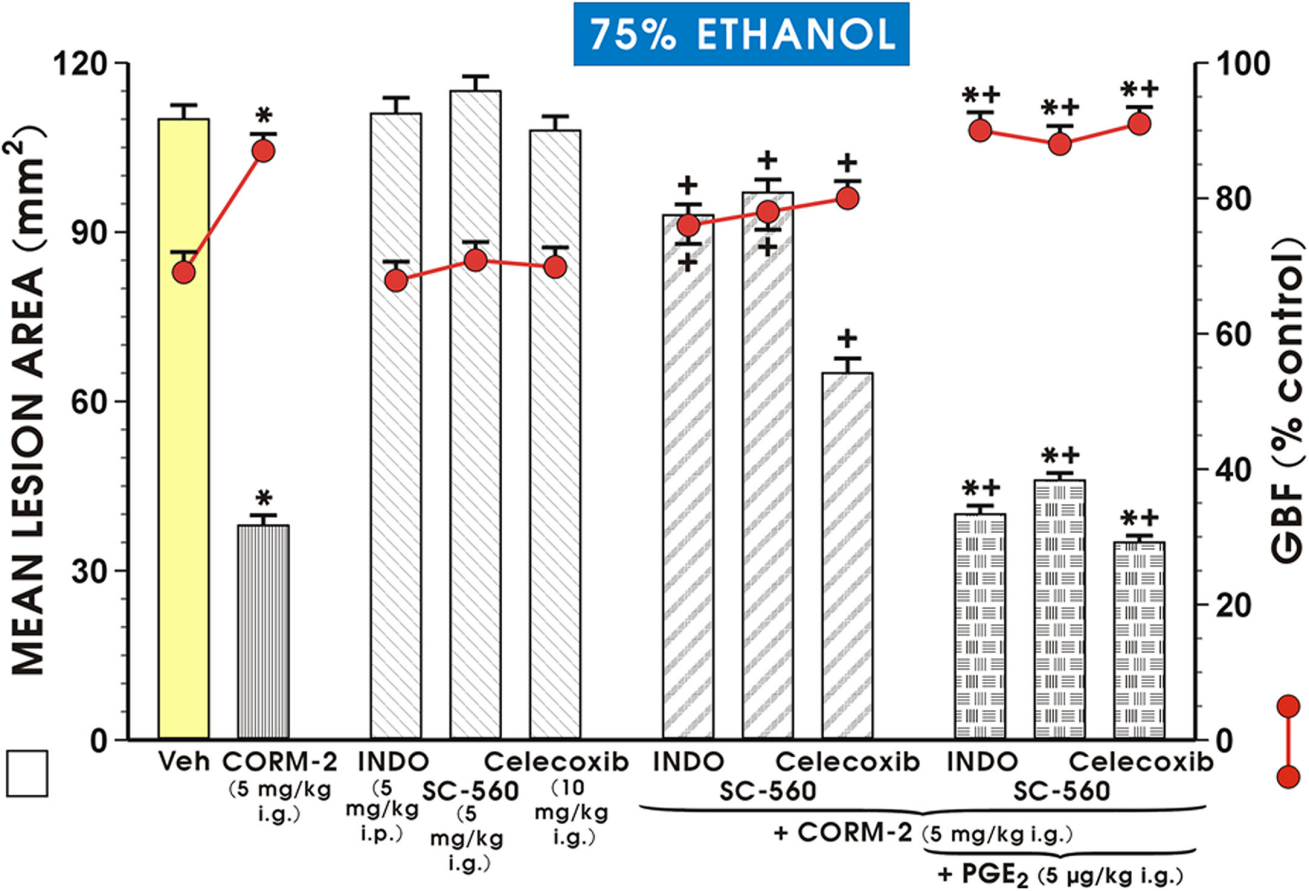
Mean lesion area of ethanol damage the alteration in the and gastric blood flow (GBF) in rats pretreated with vehicle (Veh, saline) or CORM-2 (5 mg/kg i.g) with or without the combination with a non-selective (indomethacin, Indo, 5 mg/kg i.p.) or selective COX-1 (SC—560, 5 mg/kg i.g.) or COX-2 inhibitors (celecoxib, 10 mg/kg i.g.) with or without 16, 16 dimethyl prostaglandin E_2_ (PGE_2_) and exposed to 75% ethanol. Results are mean ± S.E.M. of 7 rats per each group. Asterisk indicates a significant change (p<0.05) as compared with vehicle-control rats. Cross indicates a significant change (p<0.05) as compared with respective values in rats pretreated with CORM-2. Asterisk and cross indicate a significant change (p<0.05) as compared with rats pretreated with CORM-2 in the presence of non-selective and selective COX-1 and COX-2 inhibitors.

Fig 5 shows that the pretreatment with CORM-2 (5 mg/kg i.g.) caused a similar significant reduction in lesion area and similar increase in the GBF as presented in Figs 2 and 4. Indomethacin, SC-560 or celecoxib when administered prior to the application of 75% ethanol failed to significantly affect the mean lesion area and the GBF comparing to respective values obtained in vehicle-control group (Fig 5). However, both the reduction of ethanol lesions and accompanying increase in the GBF induced by CORM-2 (5 mg/kg i.g.) were almost completely reversed by concurrent treatment with indomethacin or SC-560 and significantly reduced by celecoxib (p<0.05) (Fig 5). These effects of the selective and non-selective COX-1 and COX-2 inhibitors co-administered with CORM-2 on lesion area and the alterations in GBF were restored when the synthetic analog of PGE_2_ (16,16 dimethyl PGE_2_, 5 μg/kg i.g.) was co-administered with CORM-2 (5 mg/kg i.g.) in the presence of COX-1 (indomethacin, SC-560) and COX-2 (celecoxib) inhibitors (Fig 5).

**Fig 6:**
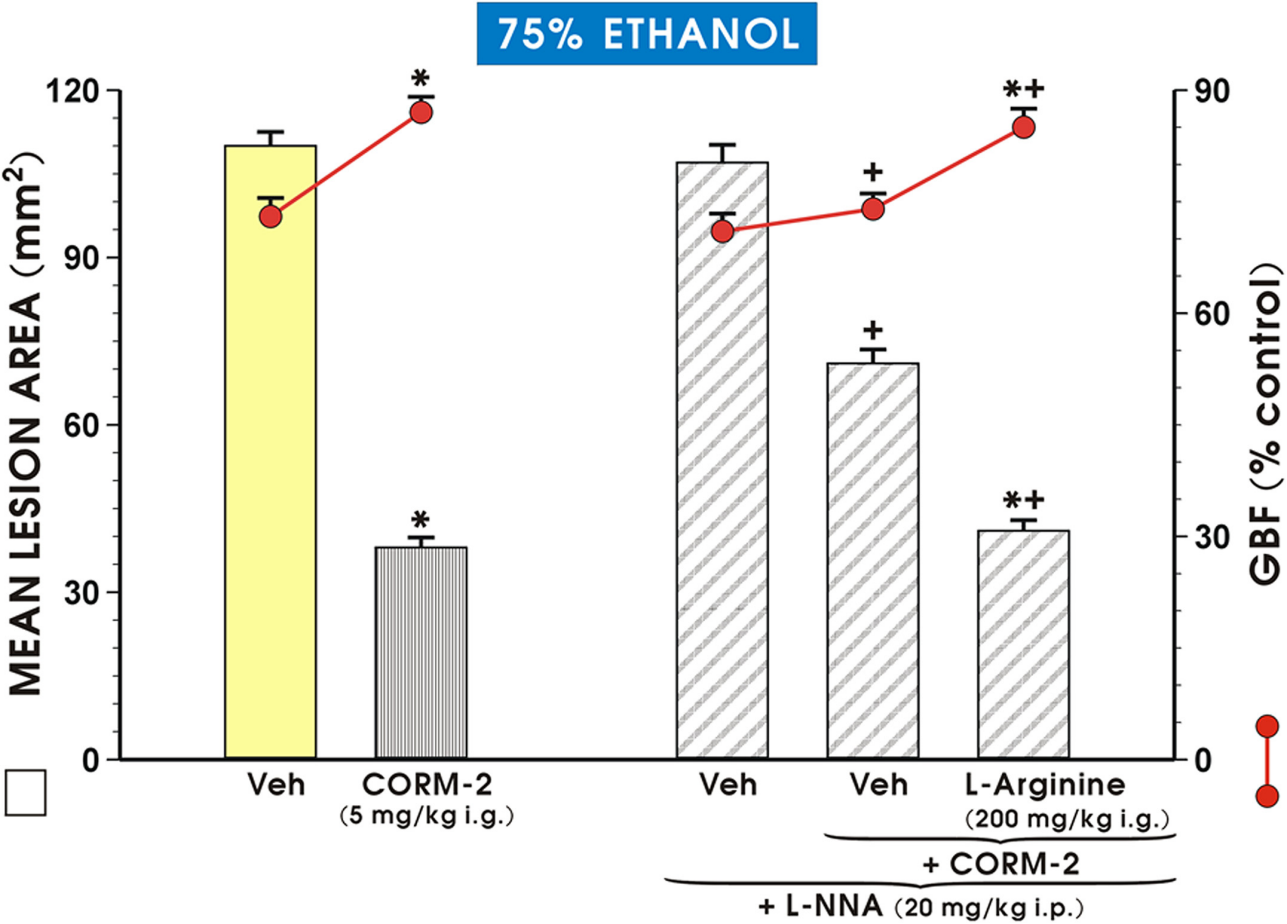
Mean lesion area of ethanol-induced gastric damage and the accompanying changes in the gastric blood flow (GBF) in rats pretreated with vehicle (Veh, saline) or CORM-2 (5 mg/kg i.g.) applied alone or in the combination with L-NNA (20 mg/kg i.p.) with or without the concurrent treatment with L-arginine (200 mg/kg i.g.). Results are mean ±S.E.M. of 6–8 animals for each experimental group. Asterisk indicates a significant change (p<0.05) as compared with vehicle-control rats. Cross indicates a significant change (p<0.05) as compared with respective values in rats pretreated with CORM-2. Asterisk and cross indicate a significant change (p<0.05) as compared with the respective values obtained in L-NNA pretreated animals.

As shown in Fig 6, the pretreatment with CORM-2 applied i.g. in a standard dose of 5 mg/kg afforded the similar reduction in area of ethanol lesions and similar increase in GBF as presented in Fig 5. L-NNA which by itself failed to significantly affect the area of ethanol-induced gastric damage and the GBF as compared with vehicle-control mucosa, but when combined with CORM-2, it significantly increased the mean area of ethanol lesions and significantly decreased the GBF comparing to the respective values obtained in rats treated with CORM-2 alone (p<0.05) (Fig 6). Concurrent treatment with L-arginine (200 mg/kg i.g.) significantly decreased the mean area of ethanol damage and significantly increased the GBF (p<0.05) comparing to rats pretreated with CORM-2 in the presence of L-NNA and exposed to 75% ethanol without L-arginine administration (Fig 6).

**Fig 7:**
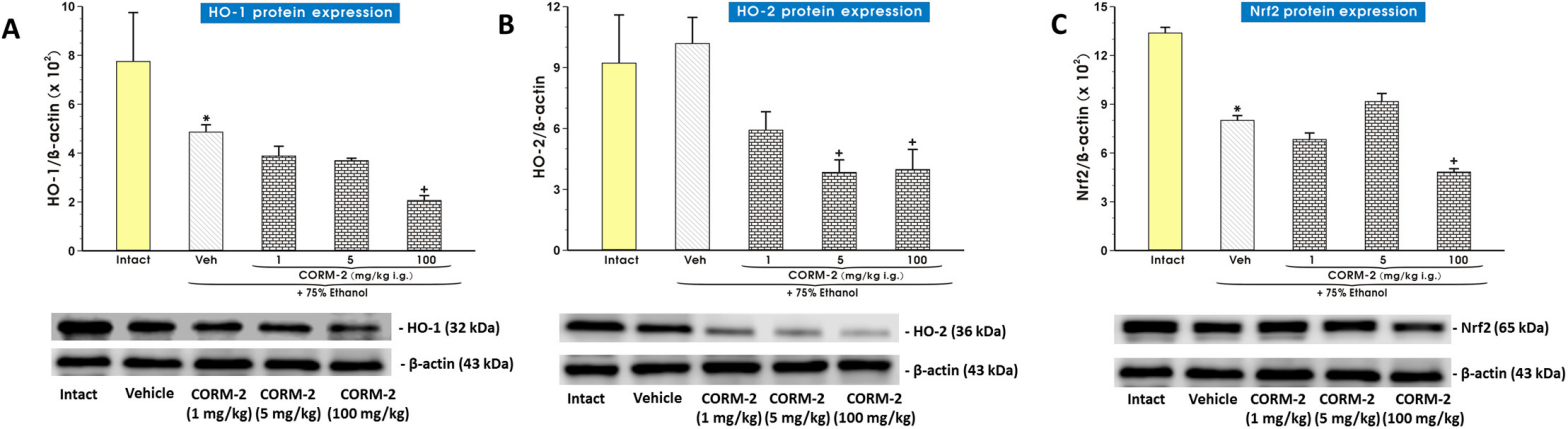
Protein expression of HO-1, HO-2 and Nrf2 in the gastric mucosa of intact rats and in those exposed to 75% ethanol with or without the pretreatment with CORM-2 applied i.g. in three doses 1 mg/kg, 5 mg/kg and 100 mg/kg. Each panel includes Western blot representative bands from each experimental group with an associated analysis of relative intensity of protein expression, normalized to β-actin expression. Panels A and C: The expression of HO-1 (A) or Nrf2 (C) was markedly decreased in the vehicle-control gastric tissue exposed to 75% ethanol as compared to intact gastric mucosa (p<0.05) as indicated by asterisk. Panels A and C: pretreatment with CORM-2 (100 mg/kg i.g.) significantly decreased the signal of HO-1 (A) and Nrf2 (C) expression as compared to those recorded in vehicle-control gastric mucosa (p<0.05) as marked by cross. Panel B: The expression of HO-2 was downregulated by CORM-2 (5 and 100 mg/kg i.g.) as compared to that received in vehicle-control group as indicated by cross (p<0.05).

Fig 7A–7C shows the effect of pretreatment with vehicle (saline) or CORM-2 applied i.g. in two gastroprotective doses of 1 mg/kg and 5 mg/kg and CORM-2 administered in high dose of 100 mg/kg which aggravated ethanol-induced gastric lesions (Fig 1) on the protein expression of HO-1, HO-2 and Nrf2 and semi-quantitative analysis of the ratio of HO-1, HO-2 and Nrf2 protein expression over β-actin protein. As shown in Fig 7A, the expression of protein for HO-1 was detectable in intact gastric mucosa. Exposure to 75% ethanol significantly reduced HO-1 protein expression in vehicle-control gastric mucosa (p<0.05) and pretreatment with CORM-2 administered in doses of 1 mg/kg and 5 mg/kg failed to significantly affect the expression of HO-1 as compared with vehicle. However, CORM-2 applied in the highest dose of 100 mg/kg significantly decreased the ratio of HO-1/β-actin protein from that recorded in the gastric mucosa pretreated with vehicle-control (p<0.05) (Fig 7A). As shown in Fig 7B, the protein expression for HO-2 was detected as a signal in vehicle-treated gastric mucosa not significantly different as compared with that in intact rats but this signal was further significantly (p<0.05) decreased by CORM-2 applied i.g. in doses 5 mg/kg and 100 mg/kg. The ratio of HO-2/β-actin protein expression confirmed that CORM-2 significantly decreased the expression of HO-2 protein (p<0.05) as compared with that assessed in vehicle-control gastric mucosa (Fig 7B). Fig 7C shows the protein expression for Nrf2 in gastric mucosa of intact rats and in those pretreated with vehicle saline or CORM-2 applied in doses of 1 mg/kg, 5 mg/kg and 100 mg/kg i.g. and exposed to 75% ethanol. The Nrf2 protein expression was detectable in the intact rats but in the vehicle-pretreated gastric mucosa exposed to 75% ethanol, the protein expression of Nrf2 was significantly reduced as compared with intact rats (p<0.05) (Fig 7C). CORM-2 applied in two gastroprotective doses 1 mg/kg and 5 mg/kg failed to affect the protein expression of Nrf2 (Fig 7C). Pretreatment with CORM-2 (100 mg/kg i.g.) significantly decreased the signal of Nrf2 expression as compared to that recorded in vehicle-control gastric mucosa (p<0.05). The ratio of Nrf2 protein expression over β-actin confirmed that expression of Nrf2 was not significantly affected in gastric mucosa pretreated with CORM-2 administered in doses of 1 and 5 mg/kg but it was significantly reduced (p<0.05) by CORM-2 applied in high dose of 100 mg/kg i.g.

**Fig 8:**
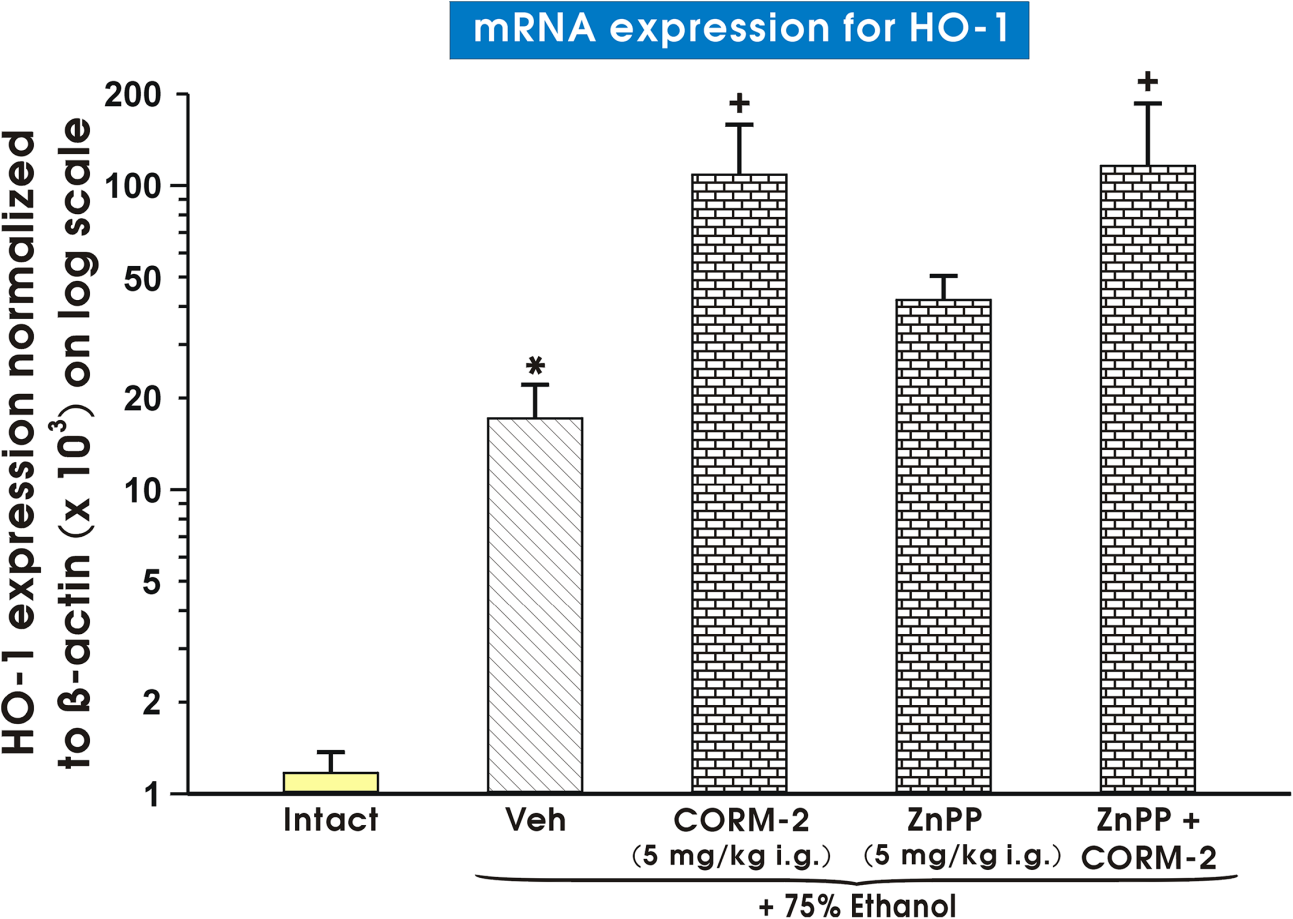
Expression of mRNA for HO-1 in gastric mucosa of intact rats or those pretreated with vehicle (Veh, saline), CORM-2 applied alone or combined with zinc protoporphyrin (ZnPPIX) and exposed to 75% ethanol. Results are expressed as the HO-1 mRNAs expression normalized to β-actin. Results are mean ± S.E.M of 4–6 determinations. Asterisk indicates a significant change (p<0.05) as compared with the respective values in intact gastric mucosa. Cross indicates a significant change (p<0.05) comparing to values obtained in Veh-control group.

Fig 8 shows the effect of pretreatment with vehicle (saline), ZnPPIX (5 mg/kg i.g.) or CORM-2 (5 mg/kg i.g.) applied alone or CORM-2 combined with ZnPPIX and exposed to 75% ethanol on mRNA expression for HO-1. The mRNA expression of HO-1 assessed by qPCR was significantly increased in gastric mucosa pretreated with vehicle and exposed to 75% ethanol as compared with intact rats (p<0.02) (Fig 8). Pretreatment with CORM-2 significantly elevated the expression of HO-1 over that pretreated with vehicle (p<0.05). ZnPPIX alone did not affect HO-1 expression as compared with vehicle-pretreated control mucosa. ZnPPIX administered in combination with CORM-2 failed to change the mRNA expression of HO-1 as compared to that analyzed in rats pretreated with CORM-2 alone (Fig 8).

**Fig 9:**
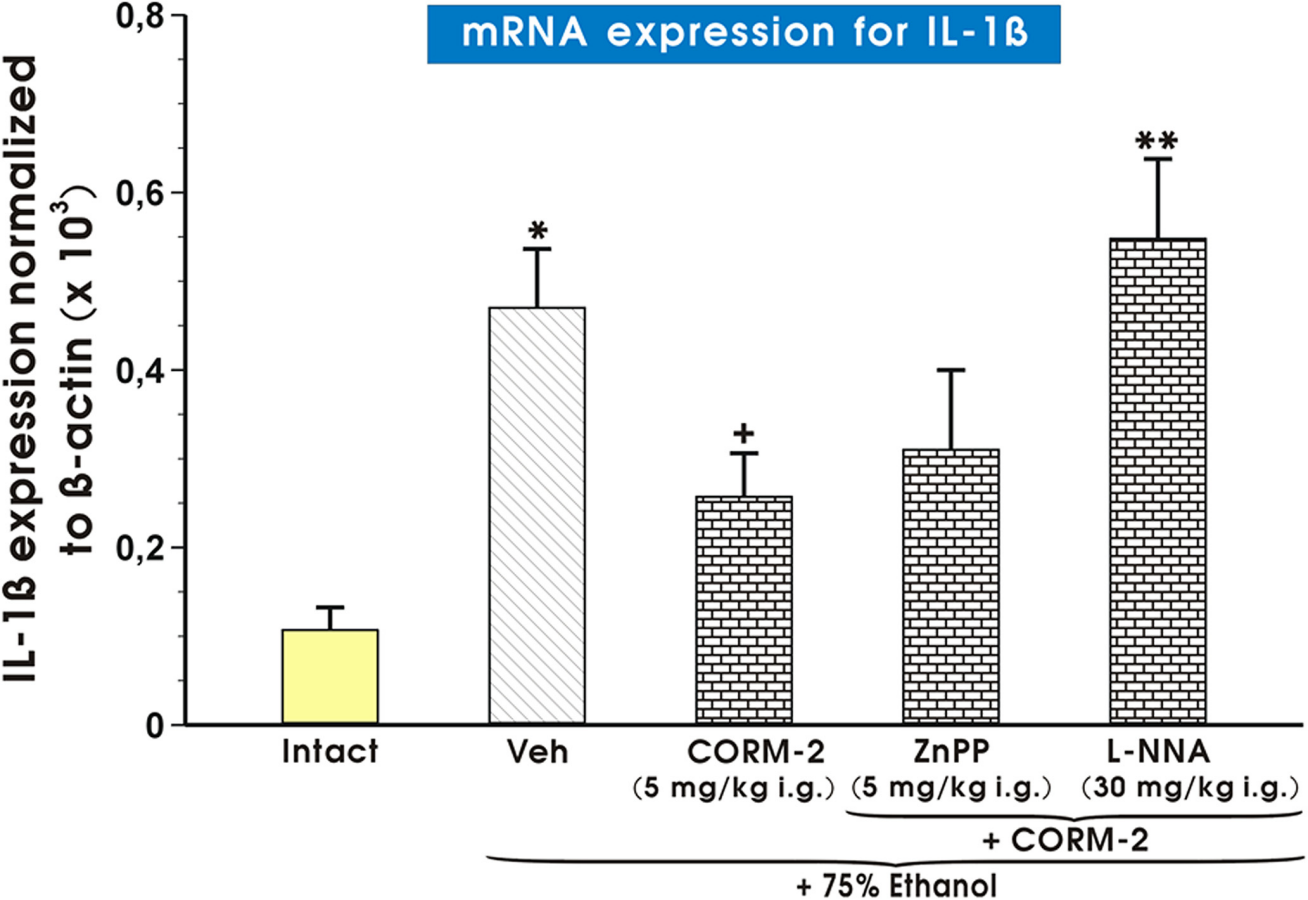
Expression of mRNA for IL-1β in gastric mucosa of intact rats or those pretreated with vehicle (Veh, saline) or CORM-2 (5 mg/kg i.g.) with or without the combination with L-NNA or zinc protoporphyrin (ZnPPIX) and exposed to 75% ethanol. Results are mean ± S.E.M of 4–6 determinations. Bar chart shows analysis of mRNA expression for IL-1β normalized to β-actin. Asterisk indicates a significant change (p<0.05) as compared with the respective values in intact gastric mucosa. Cross indicates a significant change (p<0.05) comparing to values obtained in Veh-control group. Double asterisk indicate a significant change (p<0.05) comparing to values obtained in the rats pretreated with CORM-2 alone.

As shown in Fig 9, the expression of mRNA for IL-1β was significantly increased in gastric mucosa compromised by 75% ethanol as compared with intact rats (p<0.05). In gastric mucosa of rats pretreated with CORM-2 (5 mg/kg i.g.), a significant decrease of the ratio of IL-1β/β-actin mRNA was observed as compared with that recorded in vehicle-control gastric mucosa (p<0.05). L-NNA, but not ZnPPIX, administered in combination with CORM-2 significantly decreased mRNA expression of IL-1β as compared to rats pretreated with CORM-2 alone (p<0.05) (Fig 9).

**Fig 10:**
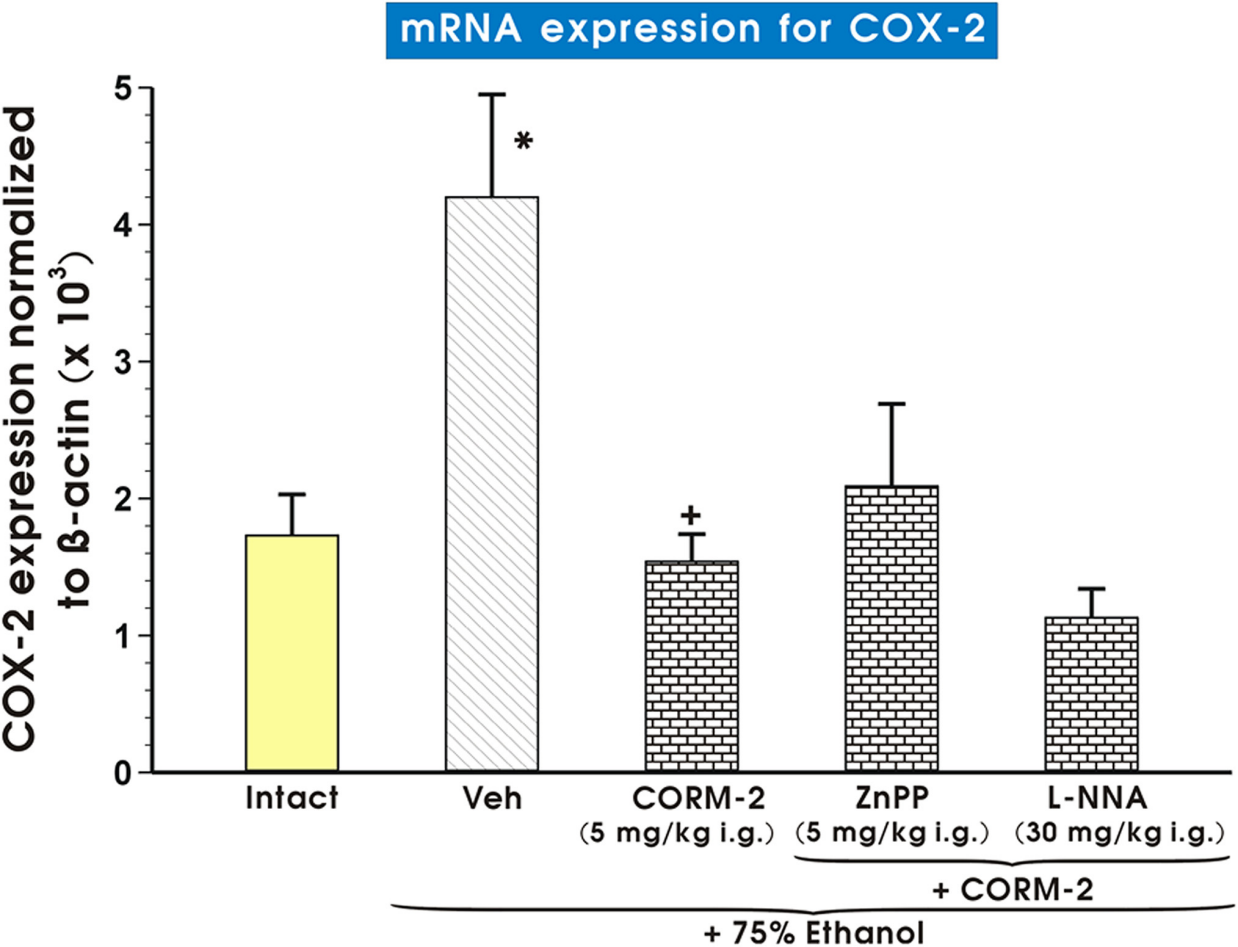
Expression of mRNA for COX-2 in gastric mucosa of intact rats or those pretreated with vehicle (Veh, saline) or CORM-2 (5 mg/kg i.g.) with or without the combination with L-NNA or zinc protoporphyrin (ZnPPIX) and exposed to 75% ethanol. Results are mean ± S.E.M of 4–6 determinations. Bar chart shows analysis of mRNA expression for COX-2 normalized to β-actin. Asterisk indicates a significant change (p<0.05) as compared with the respective values in intact gastric mucosa. Cross indicates a significant change (p<0.05) comparing to values obtained in Veh-control group. Double asterisk indicate a significant change (p<0.05) comparing to values obtained in the rats pretreated with CORM-2 alone.

Fig 10 shows the effect of pretreatment with vehicle, CORM-2 (5 mg/kg i.g.) applied alone or combined with L-NNA on the expression of COX-2 mRNA in gastric mucosa exposed to 75% ethanol. The COX-2 mRNA was significantly increased in vehicle-pretreated gastric mucosa as compared to intact rats (p<0.05). This increase in the ratio of COX-2/β-actin mRNA expression observed in vehicle-control group was significantly decreased in gastric mucosa of rats pretreated with CORM-2 alone and in those pretreated with CORM-2 combined with L-NNA or ZnPPIX (Fig 10).

**Fig 11:**
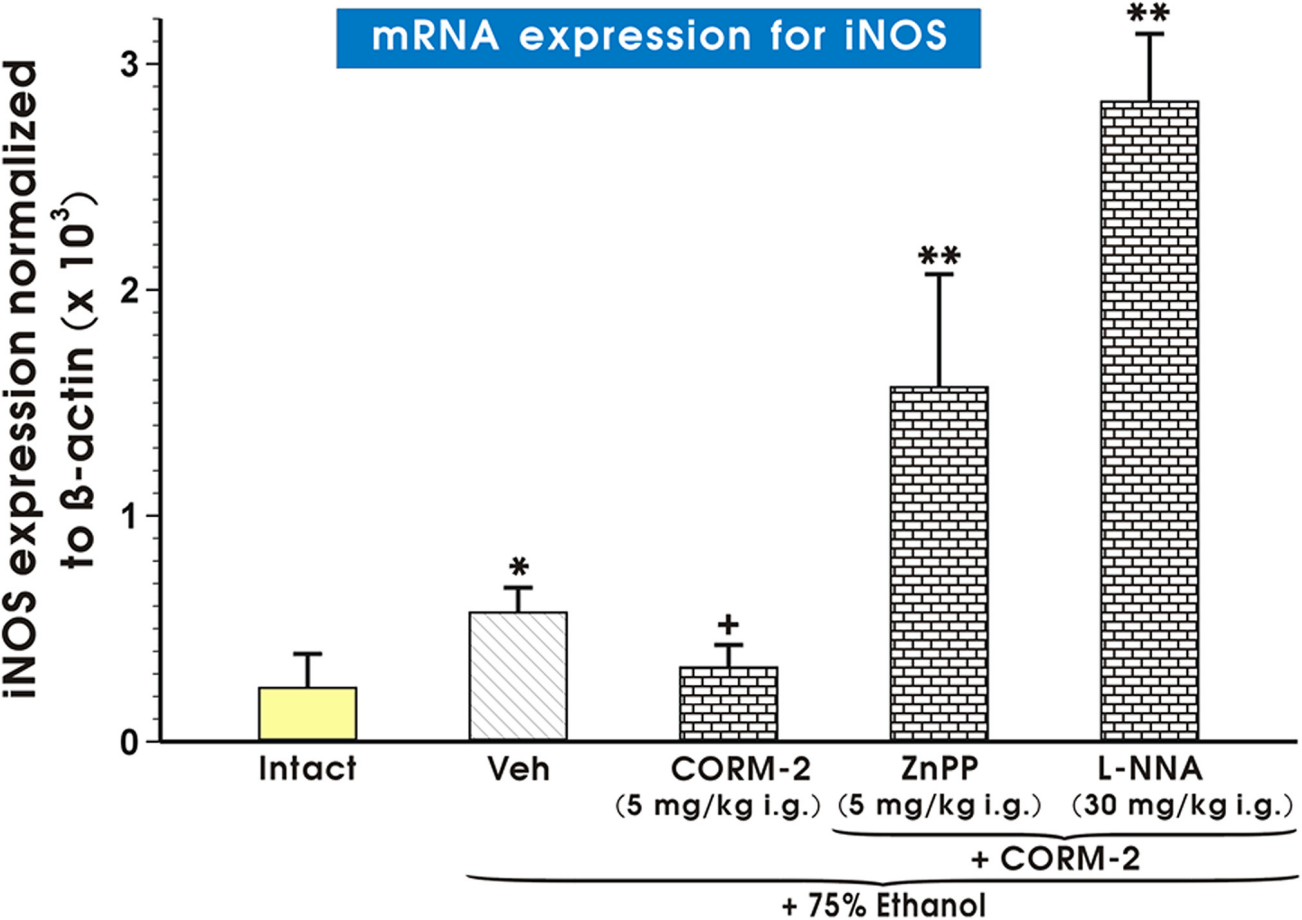
Expression of mRNA for iNOS in gastric mucosa of intact rats or those pretreated with vehicle (Veh, saline) or CORM-2 (5 mg/kg i.g.) with or without the combination with L-NNA or zinc protoporphyrin (ZnPPIX) and exposed to 75% ethanol. Results are mean ± S.E.M of 4–6 determinations. Bar chart shows analysis of mRNA expression for iNOS normalized to β-actin. Asterisk indicates a significant change (p<0.05) as compared with the respective values in intact gastric mucosa. Cross indicates a significant change (p<0.05) comparing to values obtained in Veh-control group. Double asterisk indicate a significant change (p<0.05) comparing to values obtained in the rats pretreated with CORM-2 alone.

Fig 11 shows the mRNA expression for iNOS in gastric mucosa of intact rats and in those exposed to 75% ethanol with or without the pretreatment with vehicle or CORM-2 alone or CORM-2 given in combination with ZnPPIX or L-NNA. The mRNA iNOS expression was significantly (p<0.05) increased in gastric mucosa of rats pretreated with vehicle as compared with intact gastric mucosa. In rats pretreated with CORM-2, the iNOS expression was significantly decreased as compared to vehicle-control group (p<0.05) (Fig 11). The expression of mRNA for iNOS was significantly increased in rats treated with combination of CORM-2 and ZnPPIX or L-NNA as compared to the rats pretreated with CORM-2 alone (p<0.05) (Fig 11).

## Discussion

The functional and structural integrity of gastric mucosa is maintained by complex of protective mechanisms including mucus and alkaline bicarbonate secretions from surface epithelial cells, undisturbed gastric microcirculation, PG generation, endothelial and epithelial NO release and the vasodilatory peptides such as cGRP released from afferent sensory nerve endings [33, 34]. It is anticipated that the HO/CO pathway plays emerging role in the mucosal defense system of GI tract but the mechanism underlying the gastroprotective effect of HO/CO system in the stomach exposed to irritating action of necrotizing agent such as ethanol has been little studied. It has been shown that CO prevented gastric mucosal damage induced by ethanol and nonsteroidal anti-inflammatory drugs (NSAIDs) [35] suggesting that CO could be involved in the mechanism of gastroprotection against mucosal damage induced by chemical stimuli. Herein, we demonstrated that oral gavage administration of CORM-2, a CO donor, dose-dependently attenuated ethanol-induced gastric lesions and this protective effect was accompanied by the rise in the GBF. We found that this beneficial action of CORM-2 could be solely due to release of CO from this molecule since we observed an increase in the CO content in gastric mucosa after this CO donor administered i.g. in two doses of 5 mg/kg and 50 mg/kg. Additionally, blood level of COHb, another indicator of CO release from CORM-2, was significantly increased after CORM-2 (both at 5 and 50 mg/kg i.g.) administration. This notion is supported by the finding that oral application of RuCl_3_ used in our study as a negative control to CORM-2 which does not release CO [16, 36–39], failed to affect gastric lesions and did not exert protective activity similar to that observed in case of CORM-2. Moreover, RuCl_3_ aggravated ethanol-induced gastric damage and decreased GBF as compared to respective values in vehicle-control mucosa confirming that the CORM-2-induced protection and hyperemia is not associated with the presence of Ru in the structure of this compound but could be mediated by CO release from this molecule. However, higher dose of CORM-2 (50 mg/kg) raised both, CO content in gastric mucosa and blood levels of COHb as compared with those in rats administered with the gastroprotective dose of this CO donor. Taken together, we conclude that CO released from CORM-2 exerts gastroprotection at certain range of dosages i.e. when CO bioavailability is slightly increased but high concentration of this molecule may evoke adverse effect and exacerbate ethanol-induced lesions. Using another CO donor, DMDC, Gomes et al. [40] have reported that CO which apparently had been released from this donor, ameliorated the ethanol-induced gastropathy possibly due to the antioxidant properties of this gas. In another study, the hepatoprotective effect of CO against ethanol-induced hepatic injury has been attributed to an activation of p38 MAPK signaling pathway by this gaseous mediator [41]. However, the detailed mechanism of gastroprotection against ethanol damage by CO donor such as CORM-2 including the possible interaction of this CO molecule with important cytoprotective mediators such as PG, NO, and HO enzyme isoforms, all implicated in mechanism of gastroprotection [42, 43] has not been so far elucidated.

Activities of both isoforms HO-1 and HO-2 are inhibited by metal protoporphyrins such as ZnPPIX and tin protoporphyrin IX [17, 26]. Interestingly, the HO-1 inhibitors have been shown to inhibit sGC and NO synthase (NOS) at high concentration suggesting that HO/CO system may interact with NO/NOS system [44]. In the present study, we have demonstrated that administration of inhibitor of HO-1 activity, ZnPPIX [26] not only exacerbated ethanol-induced gastric lesions but also attenuated the CO-mediated gastroprotection against ethanol injury and accompanying hyperemia (see Fig 3C). That is why we determined the effect of ZnPPIX on mRNA expression of HO-1. However, the administration of ZnPPIX failed to affect mRNA expression for HO-1 in gastric mucosa compromised by 75% ethanol. This suggests that ZnPPIX counteracting protective effect of CORM-2 against ethanol ulcerogenesis cannot be explained by inhibitory effect of ZnPPIX on HO-1 enzyme expression and the mechanism of action of this compound in the stomach injured by ethanol should be further investigated. In contrast, ZnPPIX has been shown to afford protection against hemorrhagic lesions induced by cold restraint stress through reduction of free and total acidity of gastric secretion and decreased lipid peroxidation [45]. This apparent difference in the effect of ZnPPIX against cold stress- and ethanol-induced gastric damage could be attributed, at least in part, to the different experimental models of gastric injury depending (stress) and not depending (ethanol) upon gastric acid studied in their [45] and our present study.

Herein, we revealed for the first time that endogenous PG can mediate the CORM-2-induced protection against ethanol injury and the accompanying rise in GBF. PGs are well known prototype gastroprotective mediators [42] and the continuous generation of PG helps to maintain mucosal integrity and gastric mucosal protection against ulcerogenic factors [33, 46, 47]. Moreover, we demonstrated that the non-specific COX-1 and COX-2 inhibitor indomethacin, as well as SC-560 and celecoxib, the selective COX-1 and COX-2 inhibitors, respectively, almost completely abolished, the protective and hyperemic effects of CORM-2 indicating that endogenous PG, potentially derived from the activities of both COX-1 and COX-2, are responsible for the beneficial protective effects of CORM-2 releasing CO against ethanol-induced gastric mucosal injury. PGs that are produced under physiological conditions in healthy gastric mucosa are mainly derived from COX-1 while COX-2 is considered as proinflammatory marker expressed in ulcerated gastric mucosa [48]. We presented here that the concurrent treatment with synthetic PGE_2_ analog restored the CORM-2-induced protection and hyperemia in the presence of these COX-1 and COX-2 inhibitors (Fig 5).

Using pharmacological approach in a form of sGC inhibitor ODQ we determined whether CORM-2-induced protection and the accompanying rise in GBF may involve the activation of sGC/cGMP system. Previous studies have focused on the sGC/cGMP pathway as a intracellular target of CO on vascular endothelium [49] and smooth muscle relaxation [50], modulation of neutrophils migration [51], the neurotransmission [52] and the inhibition of platelet aggregation [53]. In our present study the inhibition of sGC activity by ODQ reversed the protective and hyperemic effects of this CO donor. This data is consistent with study by Costa et al. [54] because in their report the protective effect of CO donor against bisphosphonate-induced gastric damage was mediated by an elevation of intracellular cGMP levels possibly by CO suggesting that sGC/cGMP system played a crucial role in this protection.

Both signaling molecules NO and CO can activate sGC by binding to heme moiety at active site, however, the enzymatic activity of GC is increased by about 130-fold by NO and 4.4-fold by CO [55]. NO has been shown to induce HO-1 expression and its enzymatic activity in vascular smooth muscle cells [56] and to mediate the release of free heme from heme proteins leading to upregulation of HO-1 expression [57]. On the other hand, the induction of HO-1 may counteract of inflammation by limiting proinflammatory iNOS expression and activity [11, 58]. Interestingly, the HO-1 and iNOS activities are regulated by different factors, independently from each other [44]. That is why we determined whether the inhibition of NO-synthase activity by L-NNA could affect the CORM-2-induced protection and hyperemia observed in our study. We found that L-NNA inhibited the CORM-2-induced protection and reversed the rise in the GBF evoked by this CO donor but the concurrent treatment of L-arginine, a substrate for NO-synthase [59], with CORM-2 restored this CO donor-induced protection and hyperemia. Activity of iNOS, enzyme which is considered as a proinflammatory marker, is directly inhibited by CO by binding to the heme moiety of the enzyme [60] In our study CO donor, CORM-2, downregulated the mRNA expression for iNOS which is corroborative with *in vitro* observations by Srisook et al. [61] and Sun et al. [62]. This CORM-2 inhibitory effect on ethanol damage was completely reversed when rats were concomitantly treated with the NOS activity inhibitor, L-NNA. This suggests that NO can mediate the gastroprotective and hyperemic effect of CORM-2 releasing CO against ethanol-induced gastric lesions by its anti-inflammatory activity in gastric mucosa injured by this corrosive agent.

The HO-1 enzyme plays an important role in gastroprotection [40] and a pivotal role in carcinogenesis [63]. Our finding that ethanol 75% elevated HO-1 mRNA expression is corroborative with observation by Gomes et al. [40] who demonstrated that 50% ethanol increased HO-1 mRNA expression in murine gastric mucosa. Our results seem to not contradict with the observation that acute alcohol exposure decreased the level of HO-1 mRNA transcripts in the liver [64]. These findings indicate that ethanol may regulate HO expression in an organ- or concentration-specific manner. Moreover, Kim et al. [65] reported an induction of HO-1 protein level by exogenous CO administration in human endothelial cells *in vitro*. Yan-Chang Yang at al. [66] recently suggested an increase in HO-1 mRNA in bovine aortic endothelial cells evoked by CORM-2. Hence, our finding that CORM-2 applied in dose 5 mg/kg i.g. strongly elevated HO-1 mRNA expression is corroborative with these observations. Interestingly, CORM-2 when applied in gastroprotective doses of 1 mg/kg and 5 mg/kg i.g. failed to influence HO-1 protein level when compared to decreased HO-1 expression after application of 75% ethanol, while in a high dose 100 mg/kg of this CO donor, the HO-1 protein expression was significantly reduced. This suggests that increased HO-1 mRNA is not enough to confirm the expression of the correspondent mature protein or ethanol can inhibit protein expression on the level of protein translation. The possibility that our protein of interest seems to be degraded by high concentration of ethanol giving rise to mRNA expression through a positive feedback mechanism cannot be ruled out. As mRNA is in most cases, translated into protein, we predicted that high mRNA levels would correspond to high protein levels of HO-1. However, CORM-2 applied in gastroprotective doses failed to influence the protein expression of HO-1 inhibited in ethanol-treated gastric mucosa, an effect possibly being a consequence of inhibitory action of ethanol on translation process of protein expression of these factors. Thus, our study suggests that mRNA expression of HO-1 cannot be directly correlated with its protein expression since post-transcriptional processes to the final synthesis of this native protein in gastric mucosa may be affected by injurious action of ethanol. We are aware that this inverse correlation between mRNA and proteins for HO-1 in this study could not be explained at present, and the detailed mechanism of this difference requires further investigations.

In this study we have determined that interrelationship between CO and Nrf2, a key transcription factor regulating host defense against oxidizing and inflammatory conditions [67]. This observation is in line with the findings of Chi et al. [68] and Wang et al. [69] who reported beneficial effect of CO within the brain being at least partially mediated by activation of Nrf2 pathway. We observed that exposure of rats gastric mucosa to ethanol had decreased the expression of protein for both, HO-1 and Nrf2. Indeed, recently the CO inhibition of LPS-induced inflammation in peritoneal macrophages were Nrf2 dependent at the level of mRNA and protein but only in wild mice comparing to Nrf2 knockout mice [70]. Interestingly, in our present study no increase in protein expression of both HO-1 and Nrf2 was detected in CORM-2 pretreated gastric mucosa in rats receiving ethanol. This discrepancy between our evidence in rats and previous studies in mice may be due to different species employed in both experimental models. Moreover, the protein expression of HO-1 and Nrf2 were markedly inhibited by CORM applied in supramaximal dose of 100 mg/kg, which exacerbated ethanol-induced gastric damage. This implies that HO-1 and Nrf2 are indispensable for dose-dependent CORM-2-induced gastroprotection due to their anti-inflammatory and oxidative stress inhibitory actions.

It is of interest that CO influences protein expression of constitutive isoform of HO-2. It has been already reported that the expression of HO-2 may be downregulated in the placental tissues of abnormal pregnancies [71] or in several human cell lines under hypoxia [72]. Herein, we demonstrated that HO-2 expression in gastric mucosa compromised by 75% ethanol has been decreased by pretreatment with CORM-2.

The proinflammatory cytokine IL-1β exerts profound effect on gastric pathophysiology, enhancing inflammatory response against various stimuli [73]. Previous study revealed that IL-1β mRNA is exclusively expressed in the ulcerated tissue [74]. In cytokine-stimulated Caco-2 cells CORM-2 regulates metalloproteinase-7 expression by inhibiting the IL-6 gene and downregulation of IL-8 which could be responsible for a partial resolution of ulcerative colitis and Crohn's disease *in vivo* [37]. We demonstrated that CORM-2 inhibited the inflammatory response induced by ethanol by significant downregulation of the gastric mucosal expression of IL-1β mRNA. In contrast, L-NNA combined with CORM-2 increased expression of IL-1β. This clearly indicates that NO signaling pathway is prerequisite for gastroprotective effect of CORM-2, at least in part, due to its activity resulting in downregulation of proinflammatory IL-1β expression by this CO donor. Moreover, we demonstrated that CORM-2 alone did not affect IL-4 mRNA expression (data not shown) but decreased “proinflammatory” COX-2 and iNOS mRNA expression which was increased in gastric mucosa injured by 75% ethanol. This observation suggests that CORM-2-induced protection against ethanol damage could be mediated by anti-inflammatory properties of CO released from this CO-donor.

In summary, our study demonstrates that CORM-2 prevented ethanol-induced gastric lesions by mechanism involving increase in the GBF mediated by the activation of sGC/cGMP, PG/COX and NO/NOS systems, activation of HO-1 enzymatic pathway due in part, by the anti-inflammatory properties of CO released from this donor. Understanding the mechanisms underlying the CO improvement of gastric mucosal defense may have important implications in revealing an extraordinary range of physiological functions regarding heme metabolism with reference to the mucosal defense and perhaps, the therapeutic opportunity against the formation of acute gastric lesions of upper GI tract.
